# Dynamic changes of fecal microbiota in a weight-change model of Bama minipigs

**DOI:** 10.3389/fmicb.2023.1239847

**Published:** 2023-10-20

**Authors:** Bo Zeng, Li Chen, Fanli Kong, Chengcheng Zhang, Long Chen, Xu Qi, Jin Chai, Long Jin, Mingzhou Li

**Affiliations:** ^1^Key Laboratory of Livestock and Poultry Multi-omics, Ministry of Agriculture and Rural Affairs, and Farm Animal Genetic Resources Exploration and Innovation Key Laboratory of Sichuan Province, College of Animal Science and Technology, Sichuan Agricultural University, Chengdu, China; ^2^Chongqing Academy of Animal Science, Chongqing, China; ^3^College of Life Science, Sichuan Agricultural University, Ya’an, China

**Keywords:** Bama pig, obesity, dietary restriction, gut microbiota, 16S rRNA

## Abstract

**Introduction:**

Obesity is closely related to gut microbiota, however, the dynamic change of microbial diversity and composition during the occurrence and development process of obesity is not clear.

**Methods:**

A weight-change model of adult Bama pig (2 years, 58 individuals) was established, and weight gain (27 weeks) and weight loss (9 weeks) treatments were implemented. The diversity and community structures of fecal microbiota (418 samples) was investigated by using 16S rRNA (V3-V4) high-throughput sequencing.

**Results:**

During the weight gain period (1~27 week), the alpha diversity of fecal microbiota exhibited a “down-up-down” fluctuations, initially decreasing, recovering in the mid-term, and decreasing again in the later stage. Beta diversity also significantly changed over time, indicating a gradual deviation of the microbiota composition from the initial time point. *Bacteroides*, *Clostridium sensu stricto* 1, and *Escherichia-Shigella* showed positive correlations with weight gain, while *Streptococcus*, *Oscillospira*, and Prevotellaceae UCG-001 exhibited negative correlations. In the weight loss period (30~38 week), the alpha diversity further decreased, and the composition structure underwent significant changes compared to the weight gain period. Christensenellaceae R-7 group demonstrated a significant increase during weight loss and showed a negative correlation with body weight. *Porphyromonas* and *Campylobacter* were positively correlated with weight loss.

**Discussion:**

Both long-term fattening and weight loss induced by starvation led to substantial alterations in porcine gut microbiota, and the microbiota changes observed during weight gain could not be recovered during weight loss. This work provides valuable resources for both obesity-related research of human and microbiota of pigs.

## 1. Introduction

Obesity is a major public health problem worldwide with related epidemics poses a threat to human health and quality of life (WHO, 2021). Gut microbiota is closely related to host obesity. Differences in fecal microbiota were found between obese and lean/normal humans in both European (Turnbaugh et al., 2009; Goodrich et al., 2014) and Asian (Liu et al., 2017; Yun et al., 2017). For mechanism and therapeutic purpose, diverse animal models have been used to investigate obesity and associated microbiota, typically including mouse, rat, and pigs (Lecomte et al., 2015; Chang et al., 2018; Kleinert et al., 2018). Pigs are recognized as important animal models in gastrointestinal tract studies due to having a similar anatomy and immune system to humans (Hvistendahl, 2012).

Studies on porcine gut microbiome indicated that gut microbiota (e.g., *Prevotella*) has an effect on the feed efficiency (Yang H. et al., 2017; Jiang et al., 2023). Few studies have directly compared the fecal microbiota of pigs with high and low body weights (Han et al., 2017; Oh et al., 2020). In fact, these studies are more inclined to productivity and not applicable to obesity issue. Typically, high-fat diet (HFD) or high-energy diet (HED) treatments are used to construct obesity models in animals. Pedersen et al. constructed HFD-induced obesity models on two kinds of minipigs (Ossabaw and Göttingen) and many obesity-related bacteria were identified (including *Bacteroides*, *Clostridium*) (Pedersen et al., 2013). Panasevich et al. also used Ossabaw pigs and treated with HDF for 36 weeks. They found that although obesity significantly lowered alpha-diversity of both cecal and fecal microbiota, there are difference in obesity associated bacteria with distinct predicted function between cecal contents and feces (Panasevich et al., 2018). However, shortcomings remain in these studies, i.e., relatively low number of individuals and samples, single time point (only at termination point), and no dynamic monitoring of microbiota changes during obesity process. In addition, although some studies have elaborately investigated the dynamic changes of swine gut microbiota at different ages (Ban-Tokuda et al., 2017; Wang et al., 2019; Luo et al., 2022), the time-period were from lactation to finishing (< 7 month), which is also not suitable for reference as obesity model (especially to simulate obesity of adult human).

Bama minipigs, an indigenous Chinese miniature pig breed, are widely used in biomedical research (Yang et al., 2015; Ruan et al., 2016). Studies on gut microbiota of Bama pigs are mainly focused on growth promoters and probiotics (Liu et al., 2017; Wang et al., 2021; Azad et al., 2022), and microbial research based on obesity model of Bama pig has not been reported. Here, we constructed an obesity model with relatively large number of adult Bama pigs (2 years age), and fecal microbial ecosystem was analyzed using 16S rRNA gene sequencing. We aimed to address the following objectives: (1) to dynamically delineate changes in diversity and composition structure of pig gut microbiota during weight gain and weight loss, (2) to identify gut bacteria highly correlated with pig body weight. This study has great significance not only as reference for accurately understanding the relationship between human obesity process and gut microbiota, but also to provide bioinformatical resource for future comparative research in pigs.

## 2. Materials and methods

### 2.1. Animal group design and sampling

The animal experiments were conducted with design and methods similar to those used in our previous multi-omics research project (Jin et al., 2023). A total of 60 2-year-old female adult individuals were selected from a large population of purebred Bama minipigs (a closed breeding herd which originally introduced from the national conservation farm located at Bama Yao autonomous county of Guangxi Zhuang autonomous region, China) and used in this study. All pigs were raised in the same experimental field under the same environmental conditions (Hengshu Bio-Techonlogy, Yibin, Sichuan, China). The animals were not treated with any vaccines, antimicrobials or other drugs from 1 month before and throughout the experiment period. The environment was controlled throughout the experimental period at a room temperature of 18 ~ 22°C and humidity of 30 ~ 70%. Pigs were raised in separate cages and allowed *ad libitum* to water. All pigs were fed twice daily (7:00 am, 6:00 pm) with restricted feed intake. The feed formulation and daily dosage was determined according to the nutritional requirements outlined by the Feeding Standard of Swine (NY/T 65–2004) and published by the Ministry of Agriculture and Rural Affairs of the People’s Republic of China.

**Figure 1:**
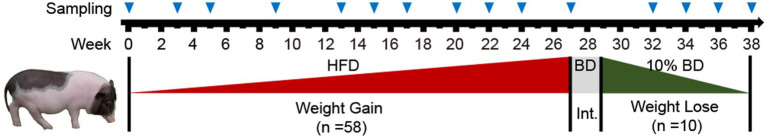
Experimental design and sampling. Animal experiments are divided into weight gain and weight loss periods, with a 2-week intermittent period (Int.) in between. Apart from the initial time point (week 0), the time point at the end of each week was used as name for labeling and describing the processing week. Bama pigs in the weight gain period (1 ~ 27) received a high-fat diet (HFD) for 27 weeks, while those in the weight loss period (30 ~ 38) were fed only 10% of a basic diet (BD) for 9 weeks. Fecal samples were collected at the time points marked by blue triangles, and body weight was recorded.

All pigs were acclimated to a basic diet (BD, 12.9 MJ/kg) for 1 week before treatment with daily dose at 3% of their initial average body weight. Then, the animal experiment was divided into a weight gain stage (Gain) and a weight loss stage (Lose) (Figure 1). During the Gain stage, pigs were fed a high-fat diet (HFD, 15.1 MJ/kg) for 27 weeks (1 ~ 27 week), and the daily dose was monthly adjusted to 3% of their current average body weight by weighing for the next month. After that, 10 pigs were randomly selected (by drawing lots) for the weight loss experiments. Pigs in the Lose stage were transferred to a separate room (still caged individually) and stabilized by feeding basic diet for 2 weeks. In order to achieve weight loss, pigs were then subjected to starvation treatment for 9 weeks (30 ~ 38 week). They continued to be fed with basic diet, but daily dose was reduced to 10% of before (0.3% of their average body weight at week 27, not changed thereafter). The above feed macronutrient content is shown in Supplementary Table S1. The pigs were observed daily to determine their health conditions, and two individuals with abnormal physical conditions were directly eliminated.

Body weight was measured regularly by moving pigs to an electronic cage scale. In order to ensure the consistency of sampling process at different time points, both the sampling time (to avoid circadian effect) and fecal sample quality were strictly controlled. Fecal samples were collected on the next day after weighing during 9:00 ~ 10:00 am with fecal container pre-cleaned the night before sampling. Feces with abnormal shape, color or volume was not included. All pigs (*n* = 58) were participated in the sampling, but only a part of individuals obtained the samples under the above conditions. Fresh feces were directly loaded into 50 mL screw-cap centrifuge tubes and immediately snap-frozen in a dry ice box, then transported to a laboratory and stored at −76°C until further analysis.

### 2.2. Sequencing and bioinformatics

Total bacterial DNA was extracted directly from each 200 mg thawed sample (scooped from the center part of feces) using TIANamp Bacteria DNA Kit (TIANGEN Biotech, Beijing, China) according to the manufacturer’s instructions. DNA concentration was measured using a NanoDrop spectrophotometer (Thermo Scientific), as well as quantified through agarose gel electrophoresis. We only selected samples from part of individuals for subsequent library construction and sequencing. Among them, all samples from 10 pigs that used for weight loss study were included, and out of the remaining 48 pigs, 30 pigs were randomly selected and their samples were sequenced (Supplementary Table S2). 16S rRNA gene amplicons were produced and sequenced at the Beijing Genomics Institute (BGI: Shenzhen, China) using the Illumina HiSeq 2 × 250 protocol. The V3-V4 region of the 16S rRNA gene was amplified using the 341f/806r barcoded primer pair (341f: 5′-XXXXXX CCT AYG GGR BGC ASC AG-3′, 806r: 5′-XXXXXX GGA CTA CHV GGG TWT CTA AT-3′).

The sequencing data analysis was performed using the QIIME2^1^ (Bolyen et al., 2019). The DADA2 method was used for sequence quality control (Callahan et al., 2016). Barcode and primer sequences were removed from the 5′ ends and low-quality bases were truncated from the 3′ ends according to the Q20 standard. The clean fasta sequences were overlapped and the feature table was constructed after de-redundancy. Features with very low sequence reads were filtered out (*n* > 30). The final high-quality representative feature sequences were used for taxonomic annotation with the SILVA rRNA database (132_99 release) used as reference^2^.

Before the diversity analysis, the feature table was collapsed to the genus level (silva taxonomy level ‘5_’) to improve biological reliability (the 16S V3-V4 region study may be not accurate at the species level). The Shannon’s diversity index and the ‘Number of Genus’ were computed for alpha-diversity, and differences between time points were tested using the Kruskal-Wallis H test with FDR based multiple comparisons adjustment (Kruskal and Wallis, 1952; Benjamini and Hochberg, 1995). Binary Jaccard and Bray-Curtis distances for each pair of samples were calculated to represent similarity relationships of the gut microbiota, and visualized using principal coordinates analysis (PCoA). PERMANOVA was used for group significance tests of beta-diversity.

Microbial composition was studied at three levels: phylum, family and genus. The relative abundances of dominant bacteria are displayed in stacked bar charts. The relative abundances data matrix at phylum and genus level were used for subsequent analysis, and low-abundance taxa (total average relative abundance <0.1%) were filtered out. The LEfSe (Linear discriminant analysis Effect Size) software was used with default parameters (factorial Kruskal-Wallis test *ɑ* <0.05, logarithmic LDA score > 2.0) to identify differentially abundant taxa between the Gain and Loss periods (https://huttenhower.sph.harvard.edu/lefse) (Segata et al., 2011). SparCC software (Friedman and Alm, 2012) was used to calculate the Pearson and Spearman correlations between bacterial taxa (genus level) and pig body weight (bootstraps, *n* = 100). The dynamic change in relative abundance of these weight-related taxa was analyzed.

PICRUSt2 (Phylogenetic Investigation of Communities by Reconstruction of Unobserved States) was used to analyze the function of the fecal microbiota (Douglas et al., 2020). The predicted metagenomes were subjected to KEGG and MetaCyc pathway analysis. Similar to microbiota analysis, the alpha and beta diversity of KEGG Orthology (KO) terms was studied and the significance of differences between groups were tested using the Kruskal-Wallis H test and PERMANOVA test, respectively.

## 3. Results

**Figure 2:**
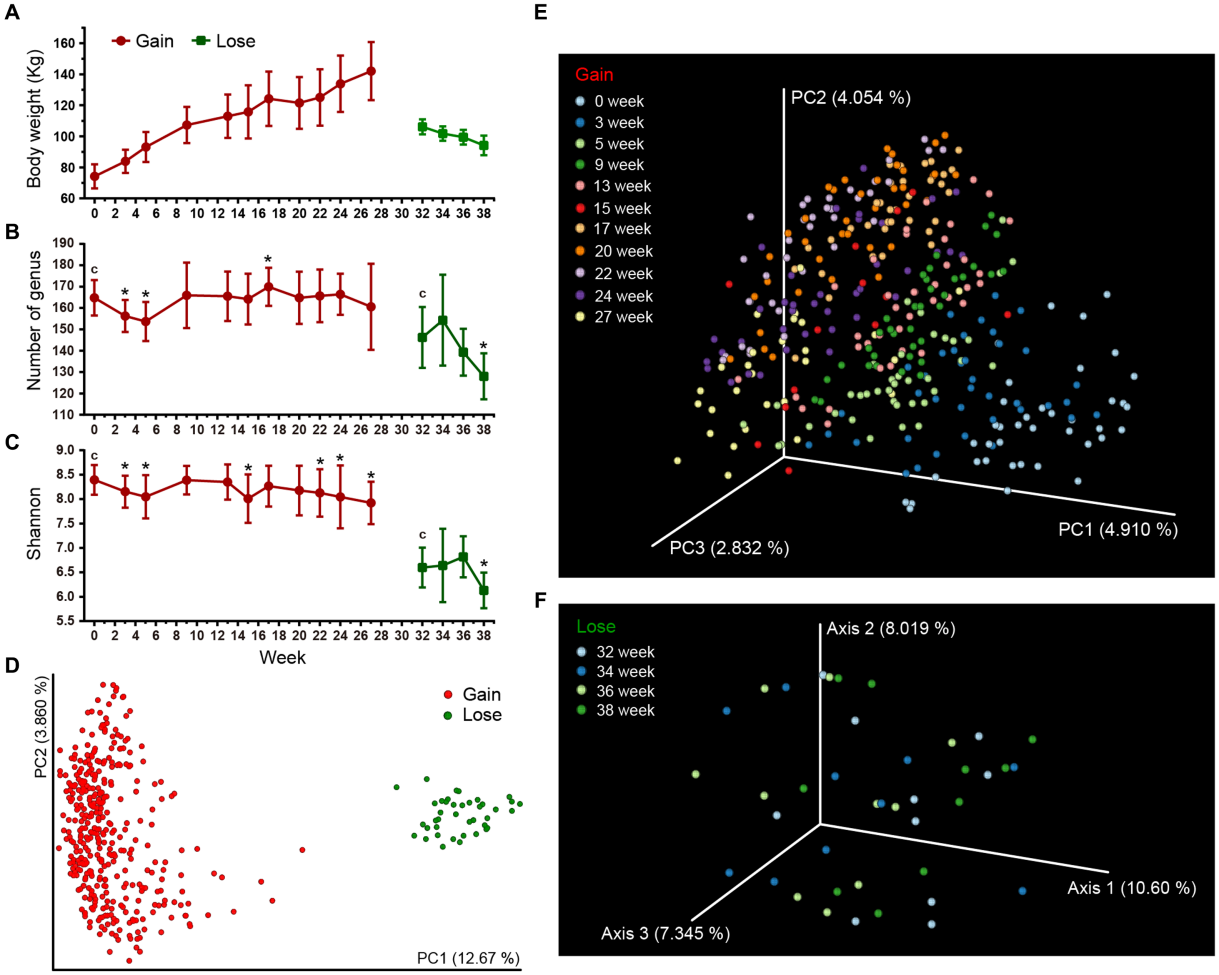
Gut microbial diversities of Bama pigs. Two different stage groups (weight gain and weight loss period) are marked with red and green colors, respectively. **(A)** Changes in the average body weight over time (only include pigs for which sequence data is available, Supplementary Table S2). **(B,C)** Alpha diversity measures of fecal microbiota using Number of Genus **(B)** and the Shannon index **(C)**. Error bars represent standard deviation (SD). Kruskal-Wallis H test was used to compare the intra-group differences between the initial time point (c) and other time points (*q < 0.05). **(D–F)** PCoA plots of beta-diversity analysis using binary Jaccard distance metrics. Each node represents the fecal microbiota of one single sample. Microbiota differences between weight gain and weight loss periods were calculated **(D)**, and the changes across time within each period are separately illustrated **(E,F)**.

The body weight of the Bama pigs changed significantly during the experiments (Figure 2A). The average weight rose from 74.2 kg at week 0 to 142.0 kg at week 27 during the Gain period. In the Lose period, it dropped from 122.2 kg at week 27 to 94.1 kg at week 38. Due to quality control of sampling and subsequent experiments (DNA extraction, library construction, etc.), a part of samples was excluded, resulting in an irregular number of samples at each time point (Supplementary Table S2). A total of 418 fecal samples were enrolled in a microbial diversity study through the 16S rRNA gene high-throughput sequencing approach, with 378 samples (13 ~ 40 per time point) in the weight gain period and 40 samples (10 per time point) in the weight loss period. After quality control of the sequence data, a total of 23,229,042 high-quality sequences were obtained with an average of 55,571 reads per sample, and the sample with the lowest sequence number has 20,636 reads. After sequence de-redundancy, we constructed the feature metadata table of all samples, which contains 7,104 high-quality features (feature min counts >30). These features were annotated and clustered into 587 taxa at the genus level, with an average of 161 genera per sample (Supplementary Table S2).

### 3.1. Dynamic changes in fecal microbial diversity

Two statistical measure, the Shannon index and the Number of Genus were calculated to study changes in the alpha diversity of pig microbiota over time. The diversity difference between initial and other time points in respective stages were compared. The high-fat diet (HFD) of the Gain period caused a “down-up-down’ fluctuation in diversity indices (Figures 2B,C). Both the Shannon index and the Number of Genus were significantly lower after 3 ~ 5 weeks of HFD treatment. By the week 9, they rose and returned to their original levels. For remaining 13–27 week, the Number of genus remained stable (one exception that increased at week 17), but the Shannon index decreased significantly by week 22 ~ 27 (Supplementary Table S3A, *q* < 0.05). After 3 weeks of starvation in the Lose period (30 ~ 32 week), the fecal microbial diversity was further sharply reduced compared to the Gain period (Supplementary Table S3A). No significant differences were found during the 32 ~ 36 week period, but by the end of the experiment at week 38, the diversity index dropped to its lowest point. In addition, we separately tracked the changes of microbial diversity of 10 individuals selected for lose period, and found that there was no significant difference from other individuals (Supplementary Figure S1), indicating that random selection had minimal impact on the diversity results.

We next assessed beta diversity with PCoA analysis and microbial composition differences of all samples were measured based on the binary Jaccard and Bray-Curtis distances of all taxa metadata. Overall, there was a significant difference between the Gain and Lose periods (Figure 2D; Supplementary Figure S2A; Supplementary Table S3B). During the Gain period, differences in microbiota between each pair of time points were all significant (Supplementary Table S3B), and the degree of distance gradually increased (compared to week 0, Supplementary Figure S3) as the Gain period continued, revealing the change of microbiota underwent a dynamic alienation process over time (Figure 2E; Supplementary Figure S2B). In the Lose period, although the overall difference among all time groups was significant (Figure 2F; Supplementary Table S3B, All between vs. All within, *p* < 0.01), there was no significant difference for pairwise comparisons between individual time groups (One exception in the Bray-Curtis results of week 34, Supplementary Figure S2C; Supplementary Table S3B). Moreover, we suspect that the microbiota changes early on during the initial 2-week period (week 30, 31) of food restriction and then stabilizes by week 32, so no gradient change similar to the Gain period occurs in the later time period.

### 3.2. Changes of the dominant gut bacteria

We analyzed changes in the relative abundance of dominant bacteria at three taxonomic levels (Phylum, Family and Genus). Overall, Firmicutes (64.3%), Bacteroidetes (27.9%), Proteobacteria (2.5%) and Spirochaetes (2.3%) were the most dominant phyla. At the family and genus level, the dominant bacteria were mainly Ruminococcaceae (29.5%, 43 taxa), Lachnospiraceae (14.6%, 64 taxa), Christensenellaceae (9.1%, 3 taxa), *Bacteroides* (3.7%), *Treponema* (2.0%), and *Lactobacillus* (1.7%).

**Figure 3:**
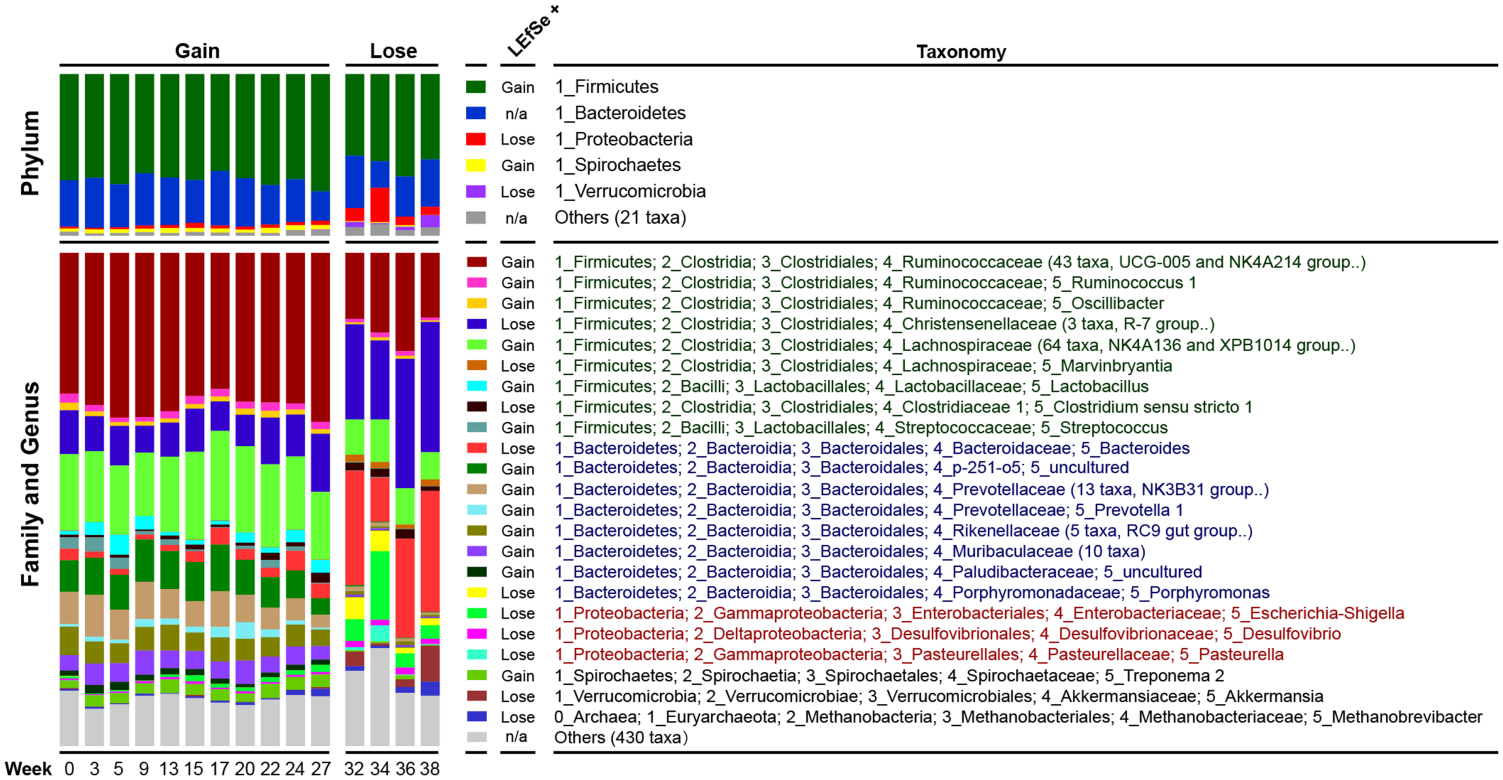
Dynamic changes in the composition of the pig microbiota. Each bar represents the average relative abundance of each taxon at a particular time point. In the ‘Family and Genus’ part, the genera with very low relative abundances (< 0.01) were summarized at the family level, and taxa that cannot be identified to the family level were merged into ‘Others’. Abundance differences of each taxon between the Gain and Lose periods were tested by LEfSe and ‘LEfSe+’ indicates the group with higher abundance. n/a, not applicable. Taxa names belonging to the same phylum were marked with the same color.

The LEfSe analysis results revealed that the gut microbiota composition underwent extensive changes between the Gain and Lose periods (Figure 3; Supplementary Table S7). Each taxon was analyzed one at a time and presented in taxonomic order at the phylum level. Firmicutes decreased in the weight loss period, mainly due to the significant decrease of taxa in Ruminococcaceae (including *Ruminococcus*, *Oscillibacter*), Lachnospiraceae, *Lactobacillus* and *Streptococcus*. Although the total relative abundance of Bacteroidetes did not change significantly, it was actually neutralized by up and down changes of taxa in genus/family level. For example, *Bacteroides* and *Porphyromonas* increased significantly in the Lose period, while the p-251-o5 family, Prevotellaceae (including *Prevotella*), Rikenellaceae, and Muribaculaceae, were all significantly reduced. Proteobacteria increased in the Lose period due to an increase in *Escherichia*-*Shigella* and *Desulfovibrio*. Furthermore, the decrease of Spirochaetes was mainly due to the decrease in *Treponema*, and increase of Verrucomicrobia in the Loss period was mostly due to *Akkermansia*.

### 3.3. Gut bacteria related to pig body weight

**Figure 4:**
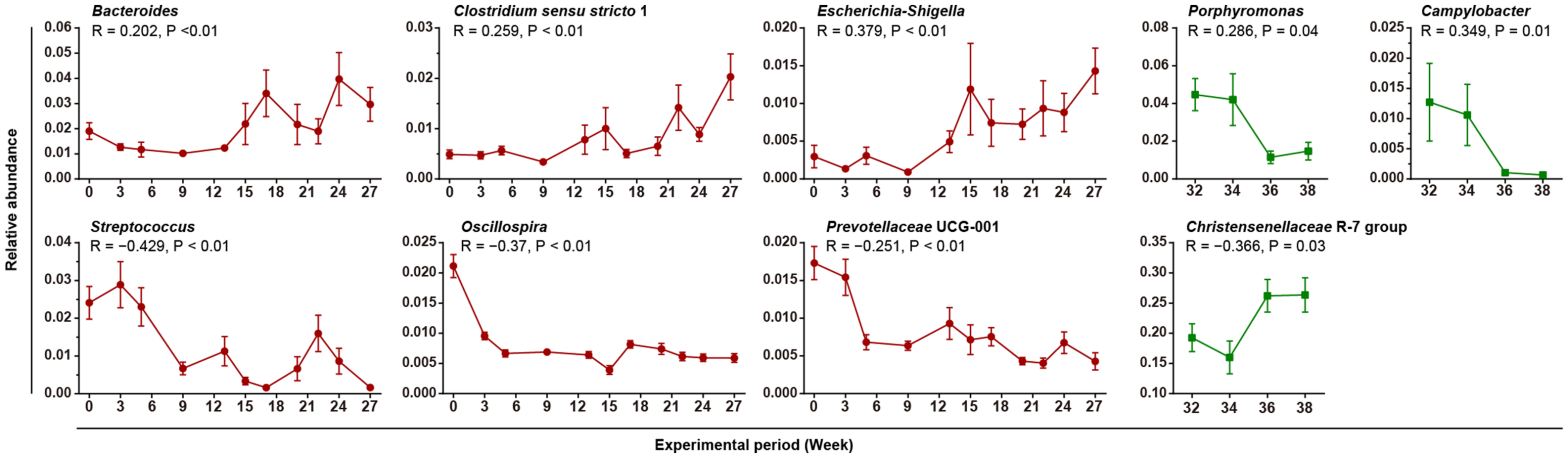
Gut bacteria correlated with body weight. Linear graphs show the average relative abundances of weight-related bacteria over time (Mean + SEM). The red and green lines indicate the Gain and Lose periods, respectively. The results of the Spearman correlation test are marked in the figure.

We looked for gut bacteria that were correlated with body weight during the Gain and the Lose periods. Only the 85 most dominant taxa at the genus or family levels (relative abundance >0.001) were enrolled in both the Pearson and Spearman correlation tests (Supplementary Table S4). After filtering (|R| > 0.2, *p* < 0.05, taxa relative abundance >0.005), 9 prominent taxa were identified, and the results were completely different between the Gain and Lose periods (Figure 4). In the Gain period, *Bacteroides*, *Clostridium sensu stricto* 1, and *Escherichia-Shigella* were positively correlated with body weight and their abundances increased over time; while *Streptococcus*, *Oscillospira* and *Prevotellaceae* UCG-001 were negatively correlated (decreased during the Gain period).

In the Lose period, *Porpyromonas* and *Campylobacter* were positively correlated and decreased in relative abundance over time (Supplementary Table S4). Only one taxon, ‘*Christensenellaceae* R-7 group’, was negatively correlated with body weight, and its average relative abundance was very high (reaching 26.3% at week 38). In addition, bacteria taxa with its abundance significant changed during weight gain period were not recovered in weight loss period.

### 3.4. Predicted function of pig gut microbiota

We next used PICRUSt2 to explore the functional profiles of pig gut microbiota. The 16S feature metadata of all samples were loaded for metagenome prediction, and predicted genes were studied for functional annotation and pathway attribution analysis based on the KEGG and MetaCyc databases. A total of 7,088 KEGG Orthology (KO) genes were acquired. The gene alpha diversity index (both Shannon and Observer KO number) was higher in the Lose group than the Gain group (Supplementary Table S5A-1, Shannon index: 10.9 vs. 10.5, KOs: ~5,384 vs. ~5,126), indicating that although starvation-related weight loss led to a significant decrease of gut microbial species, the remaining bacteria carry more types of genes and may have relatively more complex functions. Meanwhile, comparisons across time points (week groups) revealed that both alpha and beta diversity of KOs varied significantly over time (Supplementary Table S5A-2, 3, test on “week” factor).

**Figure 5:**
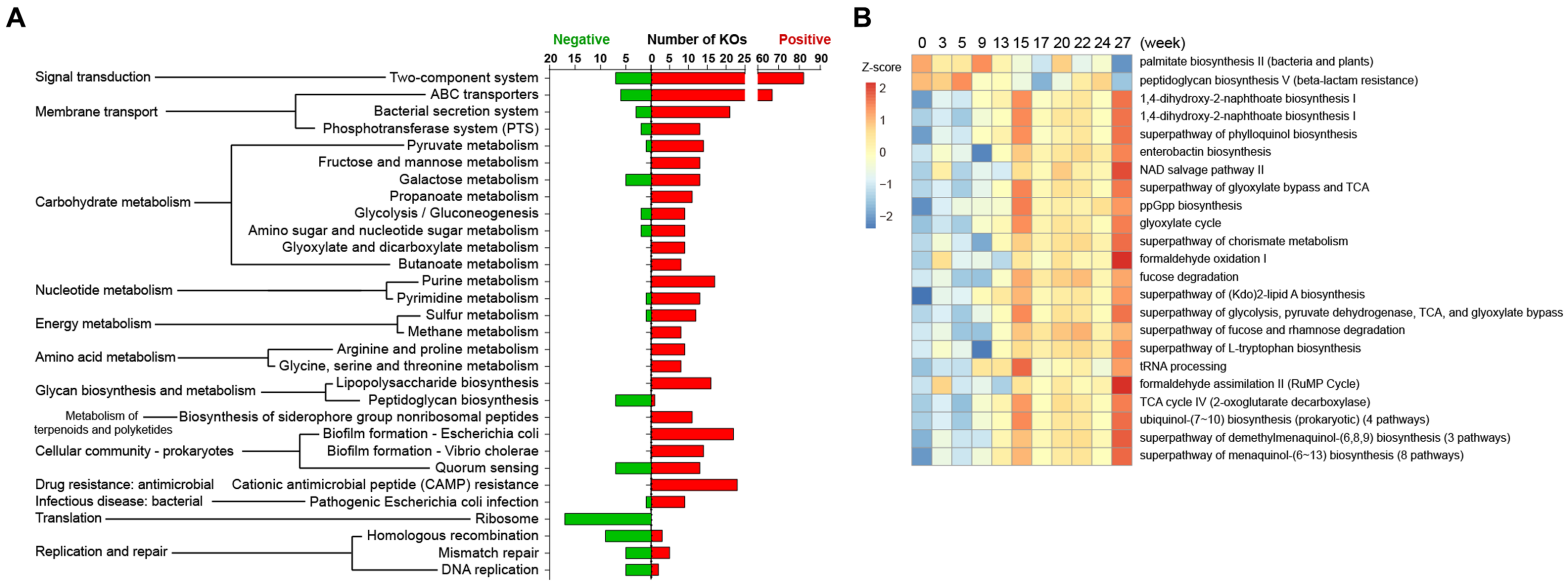
Predicted KEGG and MetaCyc pathways correlated to pig body weight in the Gain stage. Microbial metagenomic information was predicted using the PICRUSt2 package based on the 16S metadata of all samples. **(A)** KOs were tested for Spearman correlation with body weight data, and only KOs with relatively strong correlation (|R| > 0.3, *p* < 0.01, Average counts >100) were mapped to KEGG pathways. **(B)** Predicted metagenome was annotated as Enzyme Classification (EC) and mapped to the MetaCyc pathway. The aggregated enzyme gene counts data (Average counts >1,000) was used for Spearman correlation test (|R| > 0.2, *p* < 0.01), and relative abundance of these pathways were shown as a heatmap.

A correlation analysis was conducted on 4,556 abundant KOs (counts >100). In the weight gain period, 1,081 KOs were positively correlated with body weight, and 149 KOs were negatively correlated (Supplementary Table S5B). KEGG pathway affiliations of these weight-related KOs are shown in Figure 5A. However, only 6 KOs were significantly (all positive) related to body weight in the Lose period (Supplementary Table S5B), and it was not possible to find any weighted-related KOs that changed in the Gain period and recovered during the Lose period.

For the MetaCyc analysis, 2,138 predicted Enzyme genes were attributed to 415 MetaCyc pathways. Likewise, 35 pathways were corelated with body weight in the Gain period (Figure 5B, 33 positive and 2 negative) and 72 pathways were weight-related in the Lose period (Supplementary Table S5C).

## 4. Discussion

### 4.1. The influence of weight gain and weight loss on microbial diversity

The high-fat diet (HFD) treatment is commonly used to construct obesity models of experimental animals (de La Serre et al., 2010; Fei et al., 2020). Here, we do not intend to distinguish the HFD and HED (high-energy diet) in discussion, as different studies have used inconsistent ingredient and fat ratios or indeterminate energy criteria (Matteo et al., 2012; Lecomte et al., 2015; Wan et al., 2019) sIn previous studies on mice and pigs, HFD significantly reduced the alpha diversity of animal intestinal microbiota (Chang et al., 2018; Panasevich et al., 2018; Yin et al., 2018), but not significantly so in human cases, e.g., Shannon diversity was not significantly changed in young Chinese adults after a long-term HFD lasting for 6 months (Wan et al., 2019). In addition, a short-term animal-based diet (4 days, high fat and protein) also did not significantly change the alpha-diversity index (David et al., 2014). In light of these results, the influence of HFD on gut microbial diversity remains unclear. Differences in species, HFD formulations, and duration may all have impact on microbial diversity and lead to inconsistent results. In addition to HFD, the obesity traits of host may also affect gut microbiota. Several studies have shown that obese individuals are associated with higher (Kasai et al., 2015; Chávez-Carbajal et al., 2019) or lower (Turnbaugh et al., 2009; Liu et al., 2017; Yun et al., 2017) microbial diversity. HFD and the obesity phenotype are in fact two different influencing factors for gut microbiota.

In our study, the changes of fecal microbiota in pigs during long-time HFD fattening were dynamically monitored. The “down-up-down” variation of alpha diversity has never been observed before (Figure 2C) and its explanation can only be speculative. The early reduction in alpha-diversity (after 3 weeks) was mainly due to HFD treatment (consistent finding in mice, after 2 weeks) (Yin et al., 2018). The rebound of diversity at the middle time points is hard to explain, but it’s not a simple recovery, because the microbial composition has changed significantly from our beta diversity results. After a long-term HFD, excessive obesity of pigs may again lead to decrease in gut microbial diversity (consistent finding in Ossabaw pig, after 36 weeks) (Panasevich et al., 2018).

The effect of weight loss on gut microbial diversity remains controversial. In human studies, calorie-restricted diets can increase (Frost et al., 2019) or has insignificant effect (Sowah et al., 2022) on fecal bacteria diversity. Studies employing lower-fat diet (LFD) (Wan et al., 2019) and a fruit and vegetable diet (Kopf et al., 2018) were associated with increased Shannon diversity. Inconsistent reports also appeared in mouse model studies, e.g., increased (Cignarella et al., 2018) or not changed (Beli et al., 2018) in alpha diversity. Moreover, a study on obese women with very-low-calorie diets showed that although the diversity (observed ASVs) increased after diet restriction, the total bacteria copies decreased (von Schwartzenberg et al., 2021). In our weight loss experiments, we applied extreme dietary restriction (reduce 90%), a treatment that approximates the starvation model and led to a dramatic decline of alpha diversity. One study found that starvation of hybrid grouper led to significantly decreased abundance and diversity of intestinal microbiota (Liu et al., 2020). The gut bacteria diversity of brown bears was lower after hibernation, a process that may constitute a model similar to starvation (Sommer et al., 2016). Overall, we speculate that distinct weight loss strategies may results in diverse changes in microbial diversity.

### 4.2. Gut bacteria related to body weight

In the current study, *Bacteroides* was positively correlated with pig body weight in the Gain period (Figure 4, increase with weight gain) while its relative abundance still extremely increased in Lose period (Figure 3), an apparently contradictory result. HFD can cause a significant increase in *Bacteroides* in adult human (Wan et al., 2019), and research has shown that multiple species of *Bacteroides* (*B. fragilis*, *B. ovatus*, *B. vulgatus*, etc.) are positively correlated with children’s BMI (Korpela et al., 2017; Indiani et al., 2018). Interestingly, some *Bacteroides* species are negatively related to obesity and in fact are used to reverse obesity. For example, *B. acidifaciens* can prevent obesity and improve insulin sensitivity in mice (Yang J. Y. et al., 2017), and *B. thetaiotaomicron* can reduce plasma glutamate concentration and alleviate diet-induced body-weight gain and adiposity in mice (Liu et al., 2017). Therefore, we speculate that the changes in *Bacteroides* in the Gain and Lose periods in our study resulted from contributions from different *Bacteroides* species.

Multiple weight gain related bacterial taxa have also been reported in previous related studies. *Escherichia coli* was found to be higher in obese people (Gao et al., 2015; Pinart et al., 2022). A monocolonization study in mice found that *E. coli* colonization led to increased inflammation of host tissue, and aggravates HFD induced obesity and insulin resistance (Ju et al., 2023). *Oscillospira* is positively associated with leanness and health, and both HFD and inflammatory diseases can lead to a significant decrease of *Oscillospira* (Tims et al., 2013; Konikoff and Gophna, 2016; Khan et al., 2018). Similarly, the abundance of Prevotellaceae was reduced in children consuming a western-style diet (high-fat and low fiber) (Davis et al., 2020), and also in patients with urinary and nervous system diseases (Gerhardt and Mohajeri, 2018; Sanada et al., 2020; Stanford et al., 2020).

Regarding the issue of weight loss, there are variations in the changes observed in Firmicutes across different reports. In a mouse model, a significant decrease in Firmicutes was discovered when obesity was reversed through Resveratrol supplementation (Sung et al., 2017). Similar patterns have been observed in studies on hibernating animals, including grizzly bears, squirrels, and frogs, where a reduction in Firmicutes and an increase in Bacteroidetes and Verrucomicrobia were consistently observed (Dill-McFarland et al., 2014; Sommer et al., 2016; Weng et al., 2016), aligning closely with our findings in pigs (Figure 3). However, contradictory outcomes have been reported in mice subjected to intermittent fasting (Beli et al., 2018; Cignarella et al., 2018).

Christensenellaceae, as evident from our pig model study (Figures 3, 4), emerges as the most crucial bacterium associated with weight loss. It has also been found to be enriched in human individuals with a low BMI. Studies involving whole fecal bacteria transplantation (Zhou et al., 2017) and the transplantation of a single bacterium (*C. minuta*) (Goodrich et al., 2014) have demonstrated that Christensenellaceae possesses the ability to modify the composition of the microbiome associated with obesity and reduce body weight. Additionally, *Akkermansia* has garnered considerable attention (Figure 3). Consistently, fasting in mice has been shown to significantly increase the abundance of *Akkermansia* (Beli et al., 2018). Furthermore, it has been observed that both *Akkermansia* and Christensenellaceae were significantly up-regulated in a study involving the use of weight-loss drugs (quercetin and resveratrol) to alleviate obesity in mice fed a high-fat diet (Zhao et al., 2017).

### 4.3. Function of weight-related gut microbiota

In a study involving intestinal metagenomics conducted on Chinese individuals (23), it was observed that obese individuals have a lower gene count of gut bacteria compared to lean individuals. Interestingly, several obesity-upregulated KEGG pathways aligned with our findings in pigs, including ‘ABC transporters’, ‘Phosphotransferase system (PTS)’, ‘Galactose metabolism’, ‘Lipopolysaccharide biosynthesis’, and ‘Fructose and mannose metabolism’. Furthermore, two pathways, namely ‘Sulfur metabolism’ and ‘Methane metabolism’, were found to be upregulated following a high-fat diet in mice (Hong et al., 2020). Notably, the ‘Ribosome’ pathway exhibited downregulation in studies involving diabetic mice fed a high-fat diet (Liu et al., 2019) and in obese women with liver steatosis (Hoyles et al., 2018), highlighting its consistent downregulation across various obesity-related conditions. These repeatedly validated pathways warrant attention, while further investigation is needed to explore other pathways in detail.

## 5. Conclusion

The long-term HFD-fattening of Bama pigs can induce significant dynamic changes in their gut microbiota and eventually lead to a decrease in microbial diversity. The weight gain process was accompanied by an increased abundance of *Bacteroides* and *Clostridium sensu stricto* 1, and a decrease of *Streptococcus*, *Oscillospira*, and Prevotellaceae UCG-001. Weight loss through starvation did not restore the gut microbiota to its previous structure, but further reduced microbial diversity and the abundance of *Porphyromonas* and *Campylobacter*. Notably, Christensenellaceae R-7 group exhibits a significant association with weight loss. These weight-related gut bacteria in pigs could still be investigated as potential and functionally relevant microbial resources for pigs. This obesity model study based on Bama pigs revalidate many existing findings, and the original results again demonstrate the complexity of the relationship between gut microbiota and body weight.

## Data availability statement

All sequencing data are available in the NCBI Sequence Read Archive (SRA) under the bioproject number PRJNA889082, submission: SUB12136091.

## Ethics statement

All experimental procedures involving animals were performed according to Regulations for the Administration of Affairs Concerning Experimental Animals (Ministry of Science and Technology, China, revised in March 2017), and approved by the animal ethical and welfare committee (AEWC) of Sichuan Agricultural University under permit No. DKY-B20161707.

## Author contributions

LJ and ML designed the study and provided the funding support. LiC, CZ, LoC, XQ, and JC contributed to the animal experiments and sample collection. BZ performed the data analysis and wrote the manuscript. FK provided the reference analysis and constructive discussions. All authors contributed to the article and approved the submitted version.

## Footnotes

^1^ https://qiime2.org/

^2^ https://www.arb-silva.de/
