# Supplementary material for: Dynamic changes of fecal microbiota in a weight-change model of Bama minipigs
Source: Front Microbiol. 2023 Oct 20;14:1239847. doi: 10.3389/fmicb.2023.1239847 (PMC10623433; doi:10.3389/fmicb.2023.1239847)
Supplement: Supplementary file 8 [file Data_Sheet_1.docx]

Supplementary Material

Dynamic changes of fecal microbiota in a weight-change model of Bama minipigs

Bo Zeng1†, Li Chen2†, Fanli Kong3, Chengcheng Zhang1, Long Chen1, Xu Qi1, Jin Chai1, Long Jin1*, Mingzhou Li1*

*** Correspondence:** Long Jin: [longjin@sicau.edu.cn;](mailto:longjin@sicau.edu.cn;) Mingzhou Li: mingzhou.li@sicau.edu.cn

**Data availability statement**

All sequencing data are available in the NCBI Sequence Read Archive (SRA) under the bioproject number PRJNA889082, submission: SUB12136091.

**Supplementary tables**

Table S1. Dietary macronutrient content.

Table S2. Sample information and sequencing statistics.

Table S3. Significance test of alpha and beta diversities.

Table S4. Gut microbial taxa correlated to pig body weight.

Table S5. Statistical results in functional prediction analysis (PICRUSt2).

Table S6. Weight chart of all animals throughout the experiment (Kg).

Table S7. LEfSe analysis results at phylum and genus levels.

**Supplementary figures**

Figure S1. Body weight and alpha diversity of split subgroups. The pigs selected for weight loss period (n =10) were split into separate subgroups, and their data of body weight (A), Shannon diversity (B), and Number of genus (C) were separately counted and plotted. Statistical test for differences between Gain and Loss subgroups were performed in weight gain period (data of 0~24 week was used) (*p*, Mann-Whitney U test, All *vs.* All).

Figure S2. PCoA plots of beta-diversity analysis by using Bray-Curtis distance metrics. Microbiota differences between weight gain and weight loss periods was compared (A), and the dynamic changes in different time points within each period groups are separately illustrated (B, C).

Figure S3. Dynamic change of beta diversity during weight gain period. Both Jaccard (A) and Bray-Curtis (B) distance matrix data of pair-wised samples were computed and used to reflect the similarity of microbiota. The distances between initial time point and each of subsequent time points were summarized and plotted in linear graphs (Mean and SD). The "0" in the X-axis is pair-wise distance values within week 0 group, others are pair-wise distance values between compared time groups. * *p* <0.01, one-way ANOVA test among week groups of 3~27.
