# Supplementary figures and images for: Dynamic changes of fecal microbiota in a weight-change model of Bama minipigs
Source: Front Microbiol. 2023 Oct 20;14:1239847. doi: 10.3389/fmicb.2023.1239847 (PMC10623433; doi:10.3389/fmicb.2023.1239847)

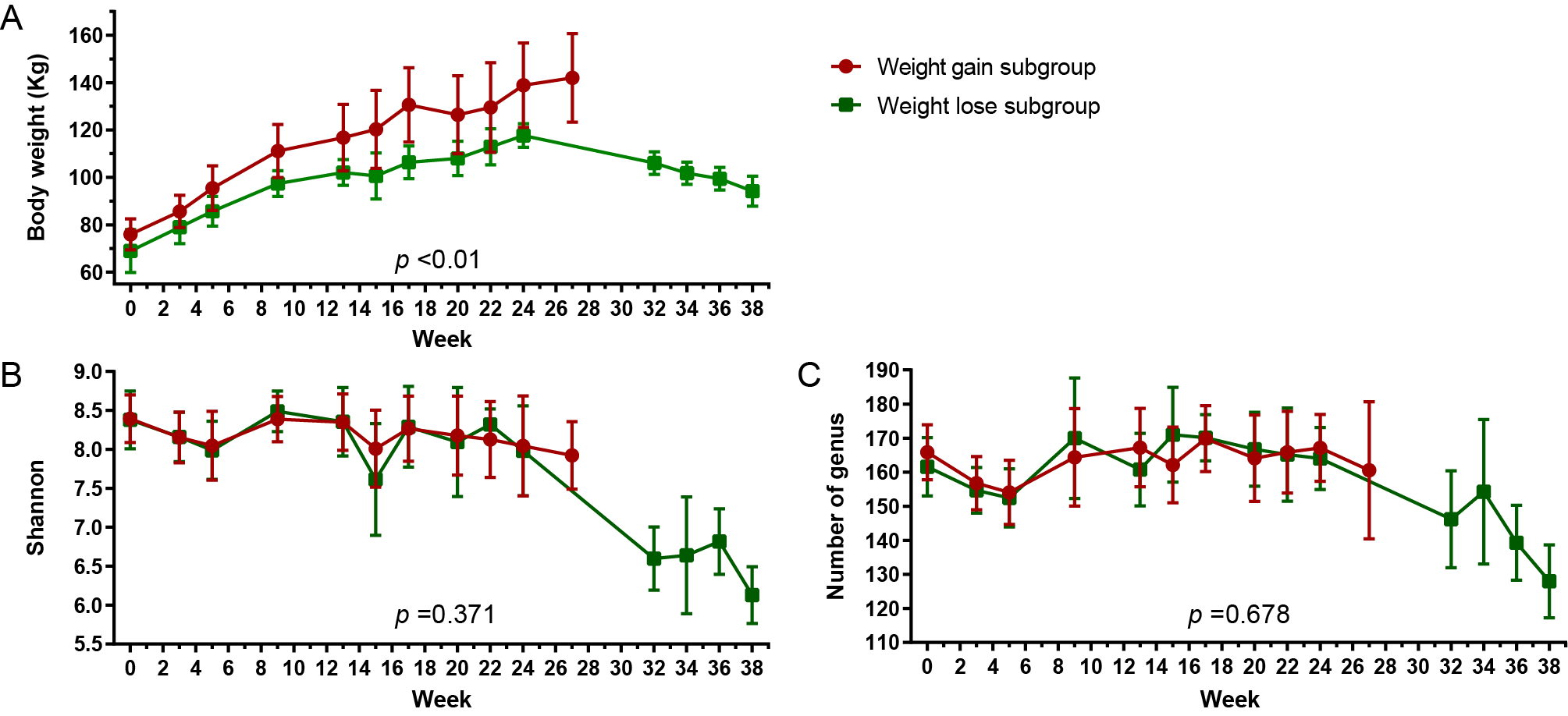

Supplement: Supplementary file 9 [file Image_1.TIF]

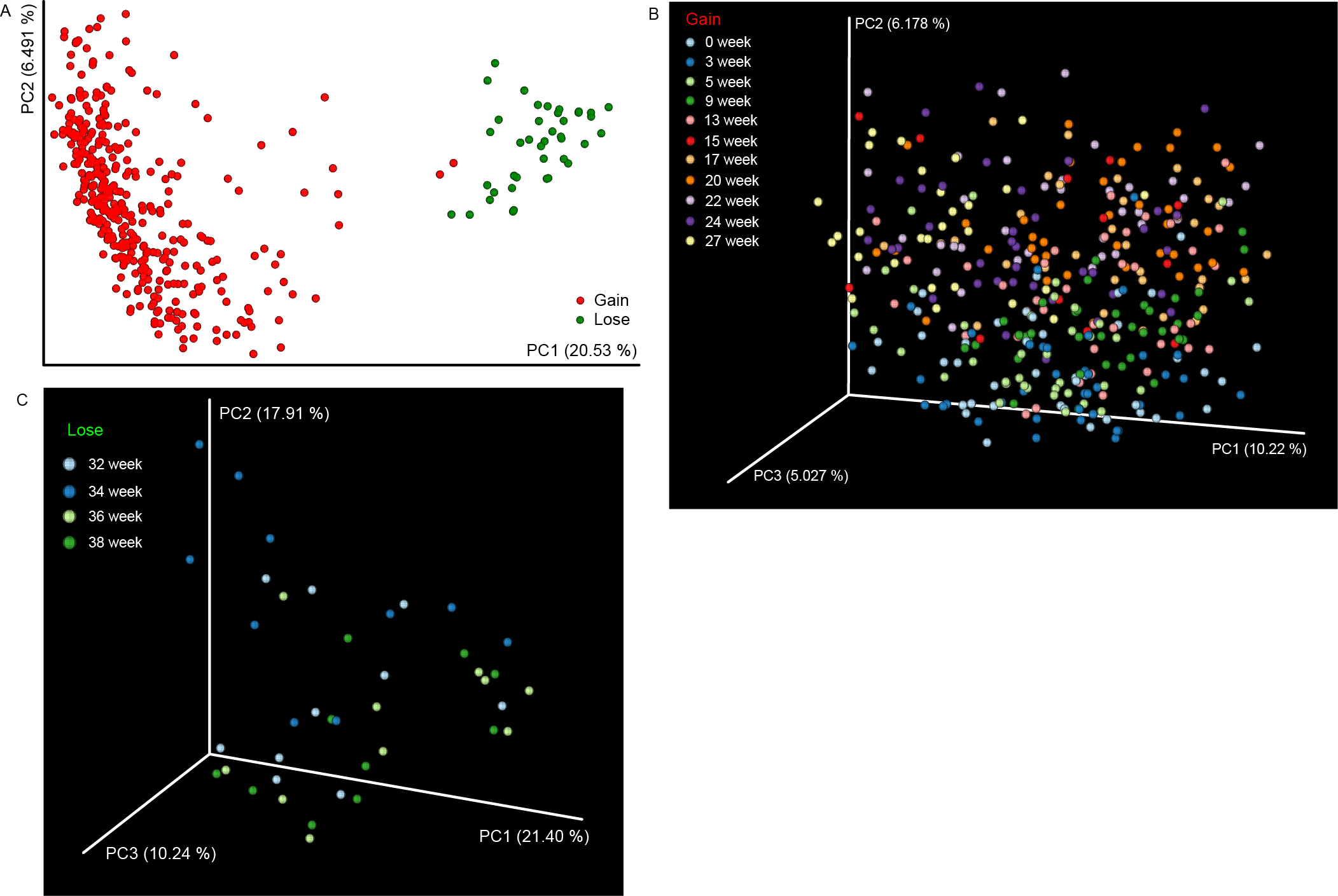

Supplement: Supplementary file 10 [file Image_2.TIF]

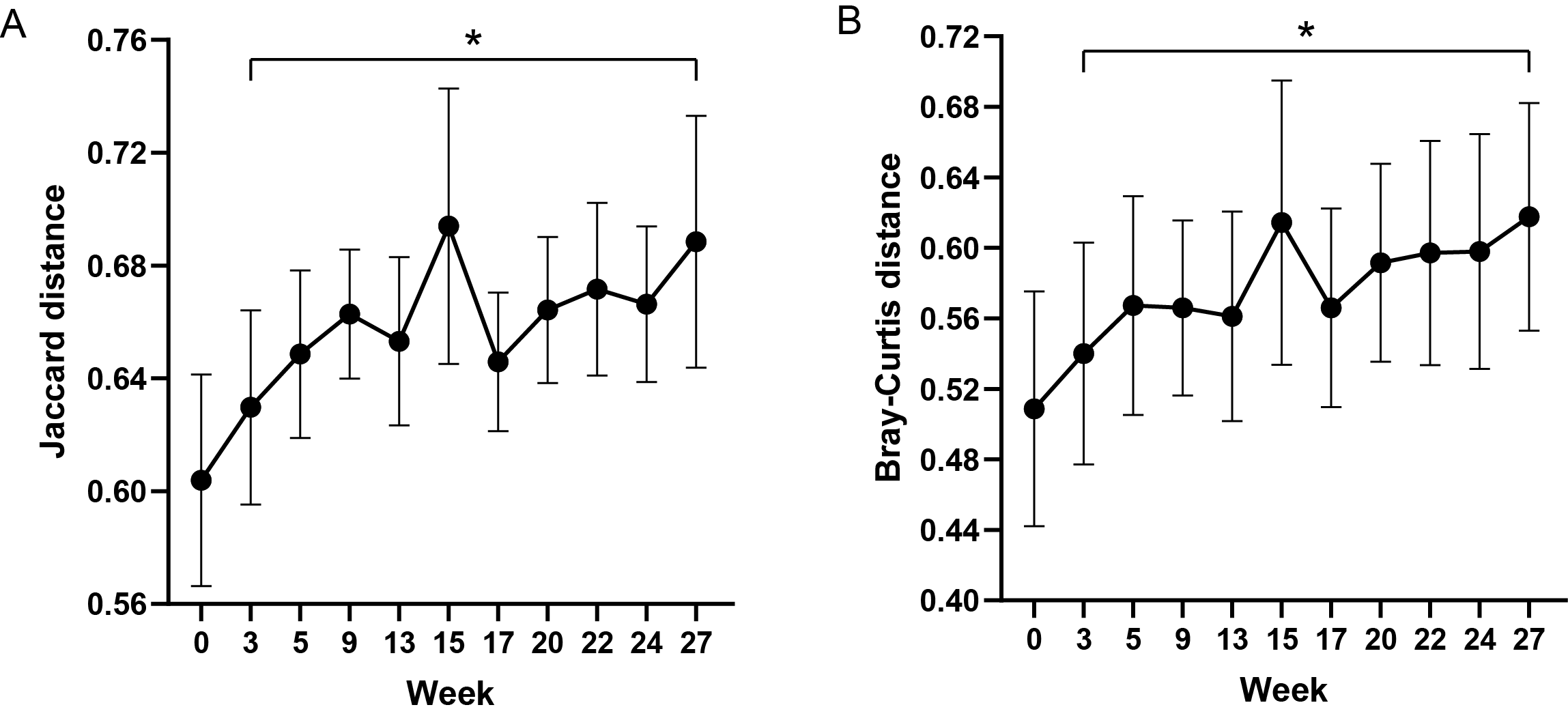

Supplement: Supplementary file 11 [file Image_3.TIF]
